# Nutritional evaluation of complementary porridge formulated from orange‐fleshed sweet potato, amaranth grain, pumpkin seed, and soybean flours

**DOI:** 10.1002/fsn3.2675

**Published:** 2021-12-16

**Authors:** Mary R. Marcel, James S. Chacha, Chigozie E. Ofoedu

**Affiliations:** ^1^ Department of Human Nutrition and Consumer Sciences, College of Agriculture Sokoine University of Agriculture Morogoro Tanzania; ^2^ Department of Food Science and Agroprocessing, School of Engineering and Technology Sokoine University of Agriculture Morogoro Tanzania; ^3^ Department of Food Science and Technology, School of Engineering and Engineering Technology Federal University of Technology Owerri Imo State Nigeria

**Keywords:** complementary feeding, iron availability, limiting nutrients, malnutrition, micronutrient density, zinc availability

## Abstract

Supplementing breastmilk with poor energy and nutrient‐dense complementary foodstuffs for young children and infants has resulted in malnutrition, poor growth, and retardation of infant development in many sub‐Saharan African countries. Ensuring nutrient adequacy for infants because of their lower consumption requires energy and nutrient‐dense food. In this context, the nutritional composition of porridge from complementary flour blends of locally available foodstuffs (orange‐fleshed sweet potato, pumpkin seeds, amaranth grains, and soybeans) was carried out. Complementary flours formulated from flour blends of pumpkin seeds, extrusion cooked soybean, and orange‐fleshed sweet potato, as well as germinated and extrusion cooked amaranth grains, resulted in varieties of complementary porridges (SAPO1–SAPO5). From these, proximate composition, mineral content (sodium, iron, magnesium, calcium, phosphorus, and zinc), vitamin contents (A and C), and nutrient density of the formulated complementary porridge were determined. Results showed that all the formulated complementary porridge were able to meet the stipulated standards of energy and nutrient (zinc, iron, vitamin A, and protein) densities. Flour blend ratio, germination process, and extrusion cooking significantly (*p* < .05) influenced the targeted nutrients of interest, as well as the nutrient and energy densities of the formulated complementary porridge. Specifically, the formulated complementary porridge with 40% amaranth grain, 25% orange‐fleshed sweet potato, 20% soybean, and 15% pumpkin seed composite mixture had 76.92% compliance level with recommended standards, which assure adequate nutrient complementation to breastfeeding. The present study provides a valuable insight that complementary foods from locally obtainable foodstuffs are potential solutions for mitigating childhood malnutrition and adequate complementation to breastfeeding by proffering the needed energy and nutrient densities required for the immunity, well‐being, growth, and development of young children and infants, without fortification.

## Graphical abstract

Specifically, the formulated complementary porridge with 40% amaranth grain, 25% orange‐fleshed sweet potato, 20% soybean, and 15% pumpkin seed composite mixture had 76.92% compliance level with recommended standards, which assure adequate nutrient complementation to breastfeeding. This study provides a valuable insight that complementary foods from locally obtainable foodstuffs are potential solutions for mitigating childhood malnutrition and adequate complementation to breastfeeding by proffering the needed energy and nutrient densities required for the immunity, well‐being, and growth and development of young children and infants, without fortification.

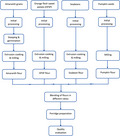

## 1 INTRODUCTION

The conventional method of breastfeeding provides the infant with several benefits such as a sufficient supply of nutrients, rapid growth, healthier and active lifestyle, and reduced risk of diseases and infant mortality (Oladiran & Emmambux, 2020). However, after 6 months of exclusive and frequent breastfeeding, infants and young children should be provided with complementary foods that are rich in energy and nutrients since their nutrient requirements can no longer be met from human breastmilk only (Agbemafle et al., 2020; Alamu et al., 2018; Ekesa et al., 2019; Tenagashaw et al., 2017; UNICEF, 2020; WHO, 2013). Complementary foods are referred to as energy and nutrient‐rich semisolid or pureed foodstuffs given to infants in addition to human milk and infant formula (Demmer et al., 2018; Kleinman, 2014). They are well known as a combined formulation of various foods developed to supply nutrients (rich in carbohydrates, lean protein, healthy fat, minerals, and vitamins) obtained from various sources including legumes, cereals, vegetables, and fruits, which are critical for healthy living of young children (Abamecha, 2020; Abeshu et al., 2016; WHO, 1998). Complementary feeding aims to ensure that in the long run, the child consumes the same well‐balanced and nutritious mixed diet of “family foods” (Demmer et al., 2018; Kleinman, 2014). This is a key step in the development of the eating behavior and affects directly the health and growth of an infant (Demmer et al., 2018; Greer et al., 2008).

On a global scale, one hundred and forty million children of less than five years are stunted (low height for age), and over forty‐seven million are still impacted with wasting (low weight for height), particularly during the period of complementary feeding (Agbemafle et al., 2020; UNICEF, 2020). The triple burden of malnutrition mostly affects Africa as a continent, with 30 countries suffering micronutrient malnutrition, undernutrition, and overweight (Development Initiatives, 2018; Low et al., 2020). Sub‐Saharan Africa experiences a micronutrient deficiency of as high as 49% among households (Emmaculate et al., 2020; Fraval et al., 2019). The most serious health challenge in developing countries is undernutrition, with Tanzania having the highest risk of undernutrition in the Eastern and Southern Africa. The main causes for undernutrition have been identified as poor feeding practices of infant and young child (Khamis et al., 2019). Interestingly, undernutrition is a major obstacle that prevents young children from reaching their full developmental potential and is responsible for at least 35% of mortality in children from developing countries (WHO, 2009). Undernutrition in infants and young children can be lessened by improving the feeding practices by providing appropriate nutrient‐rich foodstuffs (Agbai et al., 2021; Black et al., 2013; Ekesa et al., 2019; McCormick et al., 2019). Malnutrition/undernutrition is predominantly caused by inadequate dietary intake and micronutrient deficiency (Bailey et al., 2015). Importantly, the problem of malnutrition in many infants starts during or after the introduction of complementary foods, significantly contributing to an increased occurrence of malnourishment in children of less than five years (Mosha et al., 2000; Muhimbula & Zacharia, 2010). As most complementary foods in many developing African nations are predominantly poor nutritional cereal‐based foods (Dimaria et al., 2018; Pelto et al., 2003), malnutrition is inevitable as these traditional weaning foods are primarily starchy foods that are high in energy content, viscosity, bulk density, poor in protein quality, and generally low in nutrients (Eke‐Ejiofor et al., 2021). Thus, the appropriate progression from exclusive infant breastfeeding to complete utilization of complementary foods for weaning purposes can only be achieved when adequate, timely, safe, and appropriate amounts of complementary foods are provided to the young children as this promotes their good nutritional status and growth (Alamu et al., 2018; Eke‐Ejiofor et al., 2021; Ijarotimi & Keshinro, 2013; Issaka et al., 2015).

In consonance with many of the sub‐Saharan African households utilizing cereal‐based flours to prepare most of their staple foods, the micronutrient composition of these cereal‐based flours is low with higher quantities of antinutritional components (Emmaculate et al., 2020; Fraval et al., 2019). Tanzania, as one of the developing countries in Africa, depends on weaning foodstuffs that are obtained from locally available staple foods, especially cereals (Mamiro et al., 2005; Muhimbula & Zacharia, 2010; Nwosu et al., 2014), thereby causing a high rate of childhood undernutrition (Mosha et al., 2000). However, the commercial weaning foods, which are trusted to be nutritive and fortified, are usually not available in the rural areas, and where available, they are far from being accessed by many households due to their exorbitant prices (Dewey & Brown, 2003). Similarly, the availability of good sources of protein such as meat, eggs, milk, and fish is limited due to their high costs in the households of many developing countries, prompting the need to improve the nutritional content of the readily obtainable cereals (Emmaculate et al., 2020; Manary & Callaghan‐Gillespie, 2020). It is well known that agricultural practices, and climatic, socioeconomic, cultural, and ecological factors determine to a great extent the availability of foods in a given region, dietary pattern, and well‐being of the people (Caswell & Yaktine, 2013; Ofoedu, Iwouno, et al., 2021; Singh & Singh, 2017; Tandzi & Matengwa, 2020). Given this, the type of complementary foods fed to young children and infants in most rural communities in Africa is poised with intrinsic nutritional gaps. Besides insufficient nutrient density for vitamins A and C in most indigenously made complementary foods, the most limiting nutrients are calcium, zinc, and iron (Solomons & Vossenaar, 2013; Vossenaar et al., 2013). Though calcium is not a major concern because it could be obtained from breastmilk, the sources of zinc and iron are found dearth which are essential for normal growth, hematopoiesis, and neurologic and cognitive developments in infants (Krebs, 2000; Seth & Garg, 2011). Studies have shown that over 85% of complementary foods fed to infants aged 6–11 months failed to meet the WHO‐recommended nutrient density levels for zinc and iron (Ferguson & Darmon, 2007; Tenagashaw et al., 2017). The complementary foods on which these children are fed in the developing nations have been recounted to be unable to meet their nutrient requirements in both quality and quantity (Okoth, 2020; Webb et al., 2018). Due to poor quality complementary foods that contribute to undernutrition, there is an underlying need to develop nutrient‐dense complementary food that can meet the nutrient requirements of children aged 6–33 months (Okoth, 2020).

To reiterate, malnutrition is not only caused by lack of food but also by inadequate knowledge and exposure on the utilization of suitable available nutrient‐dense foods for infant feeding. Despite the efforts made by the UN’s Sustainable Development Goals and the Millennium Development Goals to eradicate (hidden and visible) hunger, malnutrition is still prevalent in most developing nations in Africa. With regard to infant feeding, the limitations of formulating complementary foods with only cereal‐based foods cannot be overemphasized as it assures nutritional imbalance, thereby giving rise to malnutrition. Though cereal‐based complementary foods form a vital source of nutrients for many of the infants in rural communities of low‐ and middle‐income states, developing a framework to help improve the nutrient density of such weaning food cost‐effectively is important. One essential approach to curb the limitations associated with the locally formulated complementary food is composting cereals with legumes and/or fruits or vegetables (Oladiran & Emmambux, 2020). In addition to composting, processing treatments and techniques have demonstrated a high promise in improving the nutritional properties of food. Moreover, multiple studies have shown that processing applications and treatment of legumes and cereals not only extend the shelf life of the product but also enhance nutrient availability, reduce antinutrients, and improve the rheological and flavor attributes of the product (Eke‐Ejiofor et al., 2021; Nwosu et al., 2019). Examples of such processing treatments may include but are not limited to extrusion cooking, soaking, pregelatinization, germination/malting/sprouting, and nonthermal processes (Chacha et al., 2021; Ofoedu et al., 2020; Osuji et al., 2019). Some of these processing treatments reduce or eliminate antinutritional factors and enhance digestibility and nutrient availability.

Furthermore, as breastfeeding cannot provide the nutrient necessities of a growing child (≥6 months), the World Health Organization recommends reducing childhood malnutrition sustainably by using available indigenous foodstuffs such as amaranth grains, to formulate energy and nutrient‐dense complementary foodstuffs that are nutritionally and hygienically adequate (Abeshu et al., 2016; Agbemafle et al., 2020; Khamis et al., 2019; WHO, 2008). Consequently, there has been an increase in research interest, especially in sub‐Saharan Africa with an emphasis on developing complementary foods from locally available materials (Okoth, 2020; Osendarp et al., 2016). Given this, exploring the available opportunities of incorporating locally available nutrient‐dense and diverse ingredients in complementary feeding to enhance the nutrient content of complementary foodstuffs in Tanzania is very fitting. Although Dendegh et al. (2021) evaluated stiff porridge from composite flour blends of African yam bean and pearl millet, Gemede (2020) evaluated complementary foods developed from pea, maize, and anchote flours, and Eke‐Ejiofor et al. (2021) reported the formulation of complementary food from a mixture of millet (malted and unmalted), African yam bean, and jack fruit flour blends; pertinent literature on porridges of complementary foods formulated from flour combinations of orange‐fleshed sweet potato, pumpkin seeds, amaranth grains, and soybeans is still scanty. Specifically, it is imperative to formulate a balanced nutrient‐dense complementary foodstuff from the aforementioned locally available foodstuffs using some processing techniques (soaking, germination, and extrusion cooking). In addition to that, the evidence of acceptable complementary porridge flour for weaning purposes, rich in nutrients, would demonstrate the value addition and potential the use of locally available foodstuffs would bring toward diversification of products in the weaning food industry. In this context, therefore, the nutritional composition of porridge from complementary flour blends of orange‐fleshed sweet potato, pumpkin seeds, amaranth grains, and soybeans was carried out. The developed product was expected to contribute to improving the macro‐ and micronutrient excellence of the complementary porridges used in Tanzania.

## 2 MATERIAL AND METHODS

### 2.1 Experimental schematic overview

**FIGURE 1:**
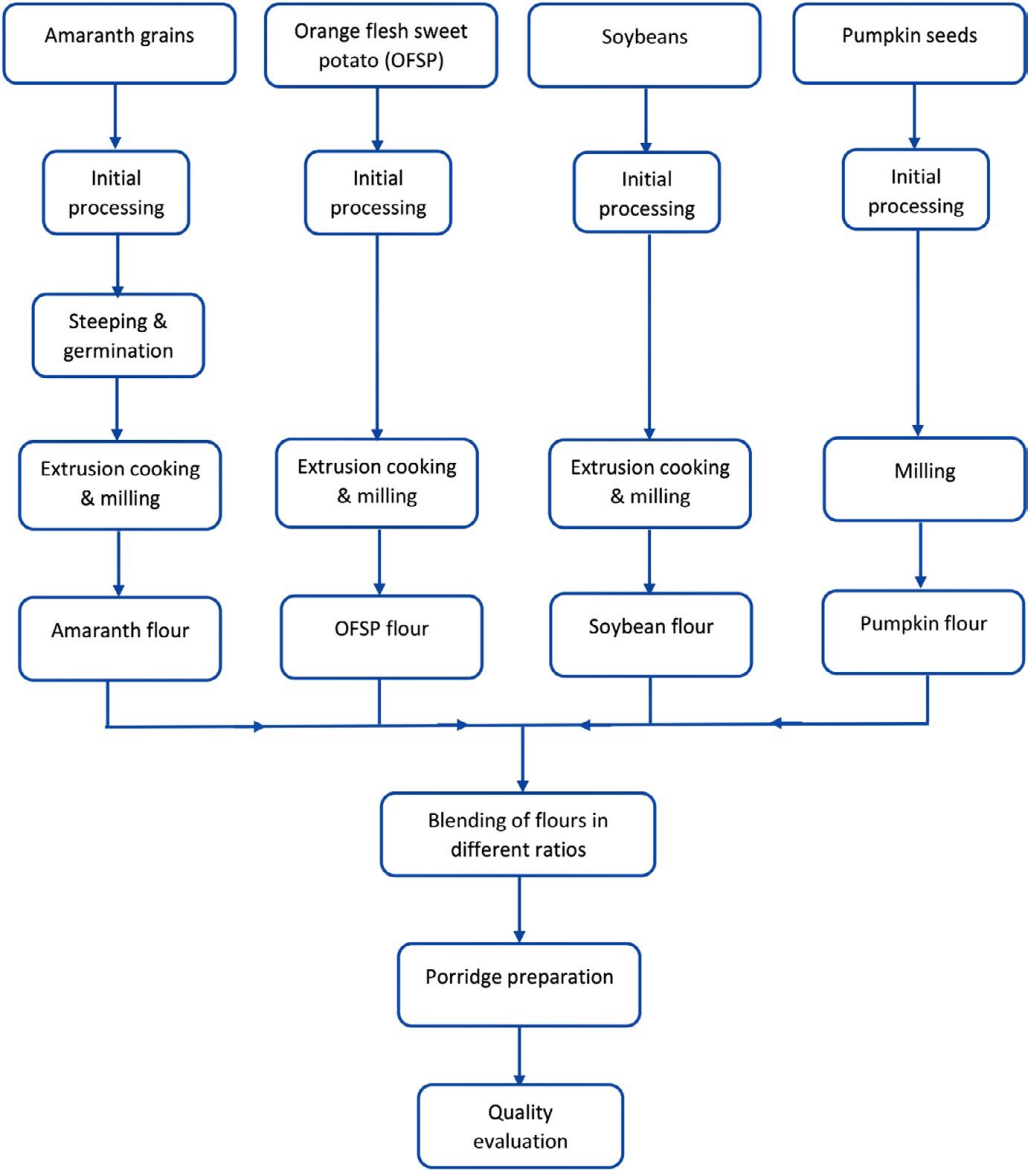
Schematic overview of the experimental program

The schematic overview of the experimental program as shown in Figure 1 presents the essential stages from raw materials to quality evaluation of the composite flours. This study was specifically designed to determine the nutritional quality of porridges from complementary flour blends of orange‐fleshed sweet potato, pumpkin seeds, soybean, and amaranth grains. Notably, two commercial formulations were used as control samples for this study. Analyses were done on porridges in duplicates by analyzing aliquot samples collected from the sample population across different blends of complementary flours.

### 2.2 Procurement of raw materials

Orange‐fleshed sweet potato, pumpkin seeds, soybean, and amaranth grains were purchased from Morogoro market, Morogoro Region, Tanzania. These raw materials were selected because of their richness in the targeted nutrients (vitamin A in the form of β‐carotene, zinc, iron, energy, and protein) as reflected in literature. The food‐grade chemicals and reagents used in this study were obtained from the laboratory of the Department of Food Science and Agroprocessing (formerly the Department of Food Technology, Nutrition and Consumer Sciences), Sokoine University of Agriculture, Tanzania.

### 2.3 Sample preparation

The raw materials (orange‐fleshed sweet potatoes, pumpkin seeds, soybeans, and amaranth grains) were processed and prepared to flour. Specifically, orange‐fleshed sweet potatoes were washed, peeled, sliced, dried in a hot air oven (Model M 30 C, S/N 92B060; Genlab, England) at temperatures between 60 and 65°C for about 3–4 h and extruded using a Twin‐Screw Extruding machine (Model Js‐60D; KY Chemical Machinery, China) followed by milling into flour using an electric blender (Blendtec FIT Model; Blendtec Inc., USA). Similarly, soybean grains were washed, boiled for about 25 min to inactivate trypsin and soften the cotyledons for easy dehulling, cooled, dehulled manually, dried in a hot air oven, extruded, and milled to flour. Also, amaranth grains were washed, steeped in water for 18 h, and germinated for 24 h to enhance digestibility and nutrient bioavailability. Subsequently, the germinated grains were washed, dried in a hot air oven, and extruded, followed by milling to flour. On the contrary, the pumpkin seeds were washed, soaked in water for 24 h to significantly reduce antinutrients, especially phytate, dried in a hot air oven, and later milled to flour. All flour samples were packaged in an airtight container (polyethylene bags) until required for use. Notably, the extrusion process was carried out under the following process conditions: 30 RPM screw speed, 10.15 kg/h feed rate, and first and second zone barrel temperatures of 100°C and 130°C, respectively.

### 2.4 Formulation of flour blends

Flours from orange‐fleshed sweet potatoes, pumpkin seeds, soybeans, and amaranth grains were blended in different proportions as presented in Table 1, to develop varieties of complementary flour of composite mixtures obtained from different levels/percentages of flour substitution. However, in this study, NutriSurvey (2007) software was utilized to design and assess complementary flour blends that have the potentials of meeting at least 50% of the recommended daily allowance (RDA) of nutrients of interest/targeted nutrients (energy: 900 Kcal; protein: 13 g; vitamin A: 300 µg; iron: 7 mg; zinc: 3 mg) for young children of age 6–12 months. Consequently, linear programming was used to optimize the flour blends that would yield an acceptable limit of the target nutrients. Subsequently, the complementary flours that met (at least) half of the targeted nutrients’ RDA were selected and progressed to porridge preparation. Therefore, from the treatment combinations, five samples were generated with the aid of the software. In other words, a combination of these ingredients (orange‐fleshed sweet potatoes + pumpkin seeds + soybeans + amaranth grains = 100%) was therefore expected to give a nutritionally balanced complementary food.

**TABLE 1:** Recipe for optimized formulation of complementary flour blends per 100g obtained using linear programming

Samples	Amount of ingredient required for composite mixture (%)				Nutritional composition of optimized diet according to the nutrient constraints				
	S	A	P	O	Energy (Kcal)	Protein (g)	Vitamin A (µg)	Iron (mg)	Zinc (mg)
SAPO1	50	20	25	5	370.2	20.7	476.7	8.6	2.8
SAPO2	40	20	30	10	393.8	22.7	869.6	8.0	4.0
SAPO3	30	35	20	15	408.1	26.7	1206.4	7.6	2.8
SAPO4	25	25	20	30	426.0	24.2	2502.2	7.9	3.4
SAPO5	20	40	15	25	349.4	28.8	2120.5	7.0	2.5

**Keys:** S: soybeans; A: amaranth grains; P: pumpkin seeds; O: orange‐fleshed sweet potato; SAPO: composite mixture of soybeans +amaranth grains +pumpkin seeds +orange‐fleshed sweet potato.

### 2.5 Porridge preparation

Five composite flours (SAPO1–SAPO5) from different blends of orange‐fleshed sweet potatoes, pumpkin seeds, soybeans, and amaranth grains (Table 1) were used to prepare porridges by mixing 350 g of flour in 1500 ml of boiling water with continuous stirring for about 15 min until the porridge was cooked. Additionally, two commercial formulations (reference/control samples) were also prepared in the same manner as described above. Importantly, the reference sample SOS was a blend of soybeans, orange‐fleshed sweet potatoes, and sorghum flours, while SMGM was a blend of soybeans, maize, groundnuts, and millet flours.

### 2.6 Quality evaluation of porridges from complementary flour

#### 2.6.1 Determination of proximate composition

The proximate composition (protein, fat, crude fiber, and moisture content) of the ingredients and the prepared porridge from complementary flours was determined according to the method described by AOAC (2012) while carbohydrate was determined by difference as shown below. To calculate the gross energy content from carbohydrate, protein, and fat contents, the conversion factors (4 kcal/g for carbohydrate, 4 kcal/g for protein, and 9 kcal/g for fat) were used (Guyot et al., 2007).

(1) \% Carbohydrate=100\%‐(Fat+\% Ash+\% Fiber+\% Protein)

#### 2.6.2 Determination of vitamin C and β‐carotene

Vitamin C content of the complementary porridge flour was determined using the titration method according to Tomohiro (1990) by titrating the extract from a mixture of flour sample (2 g) and 10% trichloroacetic acid (TCA) solution against a standard solution of 2,6‐dichlorophenolindophenol sodium salt. The amount of vitamin C content expressed as mg/100g was calculated using the formula below:

(2) $$\text{Vitamin C}\;\left(\text{mg}/100g\right)=\frac{\left(A‐B\right)\times C\times V\times 100}{D\times S}$$

where *A* is the volume in ml of the Indophenols solution used for the sample, *B* is the volume in ml of the indophenols solution used for blank, *C* is the mass in mg of ascorbic acid equivalent to 1.0 ml indophenols solution, S is the mass of sample in (g) taken for analysis, and V is the total volume of extract in milliliters

On the contrary, β‐carotene was determined using the method described by AOAC (2003), which involved homogenization of sample (5 g) in an acetone solution, followed by filtration (Whatman No. 1 filter paper; Merck KGaA, Darmstadt, Germany) of the mixture and extraction of an aqueous solution containing β‐carotene from the filtrate using petroleum spirit. A UV‐VIS Spectrophotometer Model 6305 (Bibby Scientific Ltd, Stone, UK) was employed to read the absorbance of the solution at a wavelength of 450 nm, which was computed and expressed in µg/ml β‐carotene.

#### 2.6.3 Determination of mineral content

The mineral content of the ingredients and the porridge from complementary flours blends were determined, consistent with the standard AOAC method (AOAC, 2003). This entailed initial digestion in HCl, and subsequently, the use of Atomic Absorption Spectrophotometry (AAS) Model 200A (Buck Scientific Inc., Norwalk, CT, USA) to determine the divalent cations (calcium, iron, zinc, and magnesium) of sample minerals. On the contrary, sodium and phosphorus were determined using a flame photometer (Jenway, PF 7) and colorimetric method using ammonium molybdate according to AOAC (1994).

#### 2.6.4 Determination of energy and nutrient density

The energy and nutrient density of porridges from formulated complementary flours were determined according to the methods described by WHO/UNICEF (1998) and Tenagashaw et al. (2017). Importantly, the amount of food consumed by young children (>6 months old) was estimated to be 195 ml. The energy density (expressed as Kcal/g) and nutrient density were calculated as follows:

(3) $$\text{Energy density}=\frac{\text{Gross energy of food}}{\text{Amount of food}\;}$$

(4) $$\text{Nutrient density}=\frac{\text{Targeted nutrient}}{\text{Gross energy}}\times 100$$

### 2.7 Statistical analysis

Statistical analyses were carried out using IBM SPSS software version 20 (IBM Corp., New York, USA). The assumptions of analysis of variance (ANOVA) were investigated for normality, outliers, and homogeneity of variances using kurtosis, box plot, and Levene's test. Data obtained from duplicate determinations of the sample were subjected to a one‐way ANOVA. Results of the parameters determined were expressed as mean ±standard deviation (*SD*), and the mean differences were resolved using Tukey's honest significant difference post hoc test with the significance level set at 95% (*p* < .05) confidence level.

## 3 RESULTS AND DISCUSSION

### 3.1 Proximate composition

#### 3.1.1 Proximate composition of the ingredients

The proximate composition of ingredients’ flours (soybean, amaranth grain, pumpkin seed, and orange‐fleshed sweet potato) utilized in the formulation of the complementary porridges was in the range of 2.50%–9.64% for moisture content, 2.57%–12.97% for fiber content, 2.37%–5.67% for ash content, 8.42%–34.00% for protein content, 0.69%–43.46% for fat content, and 12.16%–82.58% for carbohydrate content (Table 2).

**TABLE 2:** Proximate composition (% dwb) and total energy (Kcal/100 g) contents of soybean, amaranth grain, orange‐fleshed sweet potato, and pumpkin seed flours

Ingredients	Moisture	Protein	Fat	Fiber	Ash	Util. CHO	Gross energy
Soybean	2.50 ± 0.45^c^	34.00 ± 0.82^a^	22.30 ± 0.38^b^	6.60 ± 0.56^b^	5.67 ± 0.06^a^	29.90 ± 0.48^c^	456.30 ± 2.13^b^
Amaranth grain	9.64 ± 0.07^a^	14.75 ± 0.37^c^	8.47 ± 0.49^c^	6.56 ± 0.23^b^	2.37 ± 0.02^d^	51.64 ± 0.79^b^	341.83 ± 1.87^d^
Pumpkin seed	5.66 ± 0.04^b^	32.86 ± 0.15^b^	43.46 ± 1.08^a^	2.57 ± 0.58^c^	3.32 ± 0.07^c^	12.16 ± 0.23^d^	571.22 ± 2.45^a^
Orange‐fleshed sweet potato	5.99 ± 0.12^b^	8.42 ± 0.24^d^	0.69 ± 0.06^d^	12.97 ± 0.45^a^	4.62 ± 0.17^b^	82.58 ± 0.92^a^	370.21 ± 1.32^c^

Note

Values are means ± standard deviation of duplicate determinations.

Means with common superscripts in the same column do not differ significantly (*p* > .05)

**Keys:** Util. CHO: utilized carbohydrate

The moisture content of flour is used as an indicator of quality, since it is considered as an important factor that impacts storage, shelf life, and safety of foods (Ibeabuchi et al., 2017; Gemede, 2020). The moisture content of ingredients’ flours was significantly lower (*p* < .05) than the specified limits (14% or less) for flour moisture (Simsek, 2020). Low moisture content of flours suggests extended shelf stability as a result of prolonged drying or high drying temperature (Ibeabuchi et al., 2020; Osuji et al., 2019). However, the ash content obtained in this study from all the selected ingredients is significantly higher (*p* < .05) than the values of 2.54%–3.87% reported by Gemede (2020) for pea, maize, and anchote flours. Ash is the mineral constituent in flour (Ihediohanma et al., 2014; Nwosu, Odimegwu, et al., 2014). Thus, the highest ash content of given flours may be able to meet the minimum requirements of limiting minerals in typical locally made complementary foods. The value of fiber content of ingredients’ flours was significantly higher (*p* < .05) than 0.50% reported by Osuji et al. (2019) for rice flour, and was higher than 1.92% and 2.51% reported by Gemede (2020) for peas and maize flours, respectively. Orange‐fleshed sweet potato with a fiber content value of 12.97% appears to be the main contributor of fiber among other ingredients. Fiber is known to cleanse the digestive tract (Emebu & Anyika, 2011) and enhance bowel movement by reducing constipation (Igwe et al., 2015; Odimegwu et al., 2019). Soybean flour had the highest protein content (34.00%) followed by pumpkin seed flour (32.86%). This study revealed that soybean and pumpkin seed flours are good sources of protein (Agbemafle et al., 2020) and may be the major contributor of protein for the complementary food formulation. Thus, their utilization and consumption may be used to mitigate protein–energy malnutrition in developing countries. On the contrary, pumpkin seed flour (43.46%) had the highest fat content followed by soybean flour (22.30%). Besides enhancing the absorption of fat‐soluble vitamins such as vitamin A, fat is an important macronutrient to be considered in complementary food formulation, as it enhances the energy density of the formulated diet (WHO, 2009). Regarding the utilizable carbohydrate, results show that orange‐fleshed sweet potato (82.58%) had significantly higher (*p* < .05) utilizable carbohydrate content when compared with other ingredients. Interestingly, about 50%–80% of OFSP’s carbohydrate content exist as starch and sugar (Harahap et al., 2020), which could add sweetness and viscous flow in the complementary porridge. Orange‐fleshed sweet potato is the main energy source in the complementary food formulation. The gross energy content of the ingredients’ flours shows that pumpkin seed flour (571.22 Kcal/100 g) and soybean flour (456.30 Kcal/100 g) had higher energy content despite their lower carbohydrate content. This corroborates the report of the World Health Organization (WHO, 2009) that fat is an integral component of complementary food formulation because they increase the energy density of foods. Hence, the fact that these ingredient flours are rich in specific macronutrients implies that when combined differently, they can meet the minimum requirements or standards stipulated by regulatory agencies.

#### 3.1.2 Proximate composition of the formulated complementary porridges

The proximate composition of the formulated porridge is shown in Table 3.

**TABLE 3:** Proximate composition (% dwb) and total energy (Kcal/100 g) contents of the formulated complementary porridge

Sample	Moisture	Crude protein	Crude fat	Crude fiber	Crude ash	Util. CHO	Gross energy
SOS	7.70 ± 0.17**^b^**	1.30 ± 0.01**^e^**	2.60 ± 0.01^g^	0.10 ± 0.00**^c^**	0.10 ± 0.00**^c^**	88.10 ± 0.21^a^	381.00 ± 1.52^e^
SMGM	8.40 ± 0.21^a^	1.40 ± 0.01**^e^**	6.10 ± 0.04^f^	0.10 ± 0.00**^c^**	0.10 ± 0.00**^c^**	77.70 ± 0.26**^b^**	371.30 ± 1.67^e^
SAPO1	4.90 ± 0.43**^e^**	5.30 ± 0.01^a^	24.80 ± 0.01^a^	0.20 ± 0.00**^b^**	0.20 ± 0.00**^b^**	64.90 ± 0.44**^e^**	504.00 ± 2.35^a^
SAPO2	4.90 ± 0.37**^e^**	4.90 ± 0.01**^b^**	23.80 ± 0.01^b^	0.20 ± 0.01**^b^**	0.30 ± 0.00^a^	65.80 ± 0.35**^e^**	497.00 ± 2.41^b^
SAPO3	5.60 ± 0.42**^d^**	3.70 ± 0.01**^c^**	18.60 ± 0.06^c^	0.20 ± 0.00**^b^**	0.20 ± 0.01**^b^**	71.70 ± 0.51**^d^**	469.00 ± 1.42^c^
SAPO4	6.10 ± 0.31**^c^**	3.00 ± 0.06**^d^**	15.90 ± 0.03^d^	0.30 ± 0.01^a^	0.20 ± 0.01**^b^**	77.70 ± 0.05**^b^**	465.90 ± 1.22^c^
SAPO5	6.10 ± 0.03**^c^**	3.20 ± 0.01**^d^**	12.80 ± 0.01^e^	0.20 ± 0.01**^b^**	0.20 ± 0.01**^b^**	74.40 ± 0.40**^c^**	425.00 ± 1.89^d^
Codex standard^a^	<5	15	10–25	<3	<5	60 – 75	400 – 425

Note

Values are means ± standard deviation of duplicate determinations. Means with common superscripts in the same column do not differ significantly (*p* > .05).

**Keys:** Util. CHO: utilized carbohydrate.

SAPO: composite mixture of soybeans + amaranth grains + pumpkin seeds + orange‐fleshed sweet potato.

SMGM (control): composite mixture of soybeans + maize + groundnuts + millet flours

SOS (control): composite mixture of soybeans + orange‐fleshed sweet potatoes + sorghum flours.

^a^ CODEX CAC/GL ‐ 08 (1991) as described in FAO/WHO (1991).

The moisture content of the formulated complementary porridges was significantly lower (*p* < .05) compared with that of the control (SOS and SMGM) samples. This is thought to be contributed by the moisture content of the ingredient flours with lower moisture contents except for amaranth grains (9.64%) whereby the utilization of ingredients such as maize, groundnuts, millet, and sorghum in the control samples resulted in the high moisture content. However, the formulated complementary porridges had a moisture content within the acceptable Codex standard of <5% except for SAPO3, SAPO4 and SAPO5. The differences in flour proportion may have caused variations in the moisture content by affecting the volume of water used during porridge preparation to form gruel, thereby deviating it from the standard level. Also, the higher proportion of orange‐fleshed sweet potato in SAPO4 and SAPO5 (Table 1) may have contributed a considerable amount of starch due to its high carbohydrate content (Table 2), thus utilizing a higher volume of water in forming a gel‐like gruel during the complementary porridge preparation. Notably, the amount of water used during porridge preparation is very important to avoid over dilution and thinning of critical limiting nutrients in the complementary diet (Amagloh et al., 2013).

The protein content of the formulated complementary porridges was significantly higher compared with the control samples. This is due to the utilization of protein‐rich ingredients (soybean and pumpkin seeds) compared with the carbohydrate‐rich ingredients (maize, millet, and sorghum) that were utilized in the formulation of the control samples. Since most cereals such as maize have a low protein content (e.g., methionine, lysine, and tryptophan), cereal‐based complementary foods emanating from such ingredients should be augmented by protein‐rich (leguminous) foodstuffs to improve their nutritional significance (Kolawole et al., 2020). On the contrary, the formulated complementary porridges had significantly lower quantities of protein (<35%) compared with the Codex standard (15%). This is thought to be lowered by the incorporation of amaranth grains and OFSP, which have lower quantities of protein compared with soybean (34.00%) and pumpkin seeds (32.86%) (Table 2). The differences in the protein composition might be due to the varying proportions of the constituting ingredients (Table 1), most probably soybean and pumpkin seeds as the protein‐rich ingredients in this study (Table 2). Since this value falls below 50% of the Codex standard, which is regarded as the minimum acceptable levels (Adisetu et al., 2017; Codex, 2010), the formulated complementary porridges might be regarded as a poor source of protein. However, to overcome this deficiency in future complementary porridge formulations, there is a need to substitute amaranth grains and OFSP with ingredients that can raise the protein content to at least 50% of the recommended standard amounts. Alternatively, the proportion of the protein‐rich ingredients (soybean and pumpkin seeds) should be increased, or other foodstuffs that are considered protein‐rich be fed to the infant together with the formulated complementary porridge as a means of supplementing the deficiency. Protein is vital for the prevention of protein–energy malnutrition (PEM), which is frequently witnessed among children in emerging nations, particularly during weaning (Achidi et al., 2016; Adisetu et al., 2017). Common protein deficiency outcomes include stunting and wasting, with stunting being linked with hindered motor growth, weakened social productivity, and meager cognitive and school performance (Agbemafle et al., 2020; Bhutta et al., 2013).

The crude fat content of the formulated complementary porridges was significantly higher (*p* < .05) compared with the control samples (SOS and SMGM). As can be seen from Table 2, this was contributed by the incorporation of pumpkin seeds and soybean, which has good quantities of fats as 43.46% and 22.30%, respectively. On the contrary, the poor quantities of fats exhibited by the control samples are thought to be due to the utilization of carbohydrate‐rich ingredients including maize, millet, sorghum, and OFSP. Interestingly, all the formulated complementary porridges were within the recommended standard levels of 10%–25% (at least 51% of the standard), with SAPO1 (24.80%) and SAPO2 (23.80%) having the highest significant fat content (at least 95% of the standard) probably due to the higher proportions of soybean and pumpkin seed flours in the formulation recipe (Table 1). The significance of fats in the diets of young children and infants includes providing indispensable fatty acids, enhancing the absorption of fat‐soluble vitamins, and augmenting the dietary energy density (FAO, 2001; Obinna‐Echem et al., 2018).

Regarding the crude fiber content, although the formulated complementary porridges were rich in crude fiber compared with the control samples, they were, however, significantly lower (<10%) compared with the recommended standard level. Nevertheless, these porridges are considered to be within the Codex standard requirement (<3%). The crude fiber content of the SAPOs must have been majorly contributed by the incorporation of OFSP (12.97%) (Table 2). In a similar study, although the fiber content of the most preferred complementary foods was low (1.70%) (Laryea et al., 2018), it still met the established criteria, which require fiber content to be <5% (CAC, 2011). This is because high fiber contents result in bulky foods and induce flatulence, which is uncomfortable for infants (CAC, 2011). Moreover, because their digestive system is incompletely developed during the weaning period, digesting high fiber foodstuffs is difficult (Laryea et al., 2018). Although fiber is significant for the absorption of nutrients, as well as for the increased utilization of nitrogen, the high fiber content in complementary foodstuffs can result in not only high‐water absorption but also the displacement of energy and nutrient required for the growth of children less than 12 months (Klim et al., 2001; Obinna‐Echem et al., 2018).

Similarly, the crude ash content of the formulated complementary porridges was higher compared with that in the control samples (SOS and SMGM), which was significantly lower (less than 6%) but within the acceptable recommended Codex standard (<5%). In a similar study, a significantly higher amount of ash (2.71%) was recorded for the highly regarded formulated complementary food compared with the control samples (Lyarea et al., 2018). Another study further recorded the ash content of formulated complementary food as ranging from 1.26% to 2.31% (Olatunde et al., 2020). Ash content signifies the presence of minerals in food samples (Laryea et al., 2018; Owiredu et al., 2013), and this denotes that the formulated complementary foods in this study are potential sources of minerals (Table 5). The findings from this study reflect findings by Haque et al. (2013) that the proportion of soybean (at least 20% as shown in Table 1) resulted in high ash content of soybean flour as one of the ingredients (Table 2), which could play a key role in mineral composition of the formulations.

The utilized carbohydrate content of the formulated complementary porridges was slightly lower than the control samples. This was expected probably due to the utilization of carbohydrate‐rich ingredients in the control samples. Laryea et al. (2018) reiterate that most complementary food formulations in the African continent are cereal‐based, with mostly maize as the major ingredient that must be augmented with legumes such as soybean, groundnuts, and cowpeas to improve upon the other macronutrient components of the formulations including proteins and fat. However, when compared to the Codex standard, the formulated complementary porridges were all within the recommended levels (60%–75%) with SAPO4 exceedingly significantly higher (*p* < .05) compared with the Codex standard (Table 3). These results indicate that the formulated complementary porridges are rich sources of utilized carbohydrate possibly due to the incorporation of carbohydrate‐rich amaranth grain flour (51.64%) and OFSP flour (82.58%) (Table 2). According to da Silva et al. (2017), about 98% of OFSP’s carbohydrate content are easily digestible. Although carbohydrate plays a key contribution to the energy value in complementary foods and thus its content should be high, for infants to obtain the required energy, they should be as much digestible as possible (Olatunde et al., 2020).

The gross energy content of the formulated complementary porridges was significantly higher (*p* < .05) when compared to both the control samples and the Codex standard (>400%–425%). Besides a key contribution from the carbohydrate‐rich ingredients, this is thought to be due to the combined effect of incorporating energy‐rich nutrients OFSP (370.21 Kcal/100 g), amaranth grain (341.83 Kcal/100 g), soybean (456.30 Kcal/100 g), and pumpkin seeds (571.22 Kcal/100 g) (Table 2). As also stressed by Agbemafle et al. (2020), the higher energy content may be explained by the high‐fat content of the contributing ingredients (pumpkin seeds and soybean) used for the complementary food formulations. Since the gross energy content is at least 50% of the Codex standard, it implies that the formulated complementary porridges are acceptably rich sources of gross energy and can provide adequate energy density for the targeted group (Adisetu et al., 2017; Codex, 2010).

### 3.2 Mineral content

#### 3.2.1 Mineral content of the ingredients

The mineral composition of the ingredients is shown in Table 4.

**TABLE 4:** Mineral content (mg/100 g) of soybeans, amaranth grains, pumpkins seeds, and orange‐fleshed sweet potato flours

Ingredients	Sodium	Iron	Calcium	Phosphorus	Zinc	Magnesium
Soybean	3.00 ± 0.14^d^	16.40 ± 0.53^a^	300.36 ± 2.55^a^	695.20 ± 2.11^b^	2.70 ± 0.82^b^	258.20 ± 1.67^b^
Amaranth grain	8.00 ± 0.54^c^	13.00 ± 0.71^b^	189.10 ± 1.89^b^	322.80 ± 1.67^c^	4.80 ± 0.37^a^	219.50 ± 1.43^c^
Pumpkin seed	67.95 ± 1.50^b^	11.98 ± 0.14^c^	141.00 ± 1.31^c^	1040.80 ± 2.97^a^	1.22 ± 0.14^c^	344.60 ± 1.29^a^
Orange‐fleshed sweet potato	30.30 ± 1.11^a^	0.60 ± 0.01^d^	24.40 ± 0.82^d^	42.00 ± 1.38^d^	0.20 ± 0.01^d^	23.50 ± 0.85^d^

Note

Values are means ± standard deviation of duplicate determinations.

Means with common superscripts in the same column do not differ significantly (*p* > .05)

#### 3.2.2 Mineral content of the formulated complementary porridges

The formulated complementary porridges had significantly higher (*p* < .05) sodium concentrations when compared to both the control samples and the Codex standard (0.37 mg/100g). Among the formulated porridges, SAPOP2 had the highest sodium concentrations (269.70 mg/100g) while SAPO5 had the least amount of sodium (147.20mg/100g). Contrary to our findings, other studies indicated significantly (*p* < .05) lower sodium contents of 12.13–46.35 mg/100 g (Ndife et al., 2020) and 40.50–45.60 mg/100 g (Abolaji et al., 2019) in their complementary diets. The variations could be attributed to differences in ingredients used and the processing methods adopted. The level of sodium obtained in this study is thought to be majorly contributed by the utilization of pumpkin seeds and OFSP, which are good sources of sodium having concentrations of 67mg/100g and 30mg/100g, respectively (Table 4). It should be noted however that high sodium consumption is not good for health, both for adults and infants.

The formulated complementary porridges had significantly higher (*p* < .05) concentrations of iron when compared to the control samples. Among the formulated diets, only SAPO2 exceeded the acceptable limits of iron intake while other diets fell within the recommended dietary reference intake (DRI) of 11–18.60 mg/100 g. This might be due to the contribution made by the iron‐rich ingredients soybean (16.40 mg/100g), amaranth grains (13.00 mg/100 g), and pumpkin seeds (11.98 mg/100 g) (Table 4). Similar findings were recorded in a different study whereby the iron content of all the complementary formulations were at least 50% of the required standard. It is thought that this was contributed by soybeans, cowpea, and broken rice fractions containing iron‐rich bran (Adisetu et al., 2017). However, the iron bioavailability in the formulated diets might have been enhanced by the processing methods (germination and extrusion cooking) utilized during complementary food formulation by reducing or eliminating the phytate levels (Adisetu et al., 2017; Hurrel, 2004). Iron is essential in an infant diet for hemoglobin synthesis and their mental and physical welfare as its deficiency adversely impacts the growth of infants during the weaning period (Laryea et al., 2018).

All the formulated complementary porridges had significantly higher (*p* < .05) calcium contents than the control SOS (37.50 mg/100 g). However, the formulated complementary porridges SAPO1 (70.50 mg/100 g), SAPO2 (83.80 mg/100 g), and SAPO3 (63.50 mg/100 g) had significantly higher calcium contents while SAPO4 (46.40 mg/100 g) and SAPO5 (49.60 mg/100 g) had significantly lower amounts when compared to the control sample SMGMP (58.20 mg/100 g) (Table 5).

**TABLE 5:** Mineral content (mg/100g) of the formulated complementary porridge

Sample	Sodium	Iron	Calcium	Phosphorus	Zinc	Magnesium
SOS	102.70 ± 2.66^g^	6.50 ± 6.204^e^	37.50 ± 2.37^g^	157.10 ± 3.00^e^	4.10 ± 1.06^e^	100.20 ± 2.06^f^
SMGM	116.90 ± 2.35^f^	10.90 ± 2.44^d^	58.20 ± 1.19^d^	148.60 ± 5.85^f^	5.30 ± 1.71^d^	92.90 ± 2.58^g^
SAPO1	153.50 ± 7.91^d^	18.40 ± 5.46^b^	70.50 ± 3.86^b^	301.60 ± 4.07^a^	9.50 ± 2.74^b^	186.60 ± 3.86^c^
SAPO2	269.70 ± 4.82^a^	24.20 ± 2.78^a^	83.80 ± 2.93^a^	259.30 ± 2.88^d^	11.30 ± 1.86^a^	218.70 ± 3.32^b^
SAPO3	253.70 ± 2.93^b^	18.40 ± 2.99^b^	63.50 ± 4.64^c^	292.40 ± 4.30^b^	12.10 ± 1.17^a^	231.00 ± 3.46^a^
SAPO4	193.20 ± 3.36^c^	15.20 ± 2.03^c^	46.40 ± 2.62^f^	157.90 ± 2.60^e^	7.30 ± 1.58^c^	176.70 ± 2.18^d^
SAPO5	147.20 ± 4.45^e^	13.40 ± 6.37 cd	49.60 ± 3.08^e^	285.70 ± 3.13^c^	7.10 ± 0.32^c^	168.20 ± 5.37^e^
DRI^a^ (6–12 m)	0.37	11–18.60	260–400	275	3–8.40	54–75

Note

Values are means ± standard deviation of duplicate determinations.

Means with common superscripts in the same column do not differ significantly (*p* > .05).

**Keys:** SAPO: composite mixture of soybeans + amaranth grains + pumpkin seeds + orange‐fleshed sweet potato

SMGM (control): composite mixture of soybeans + maize + groundnuts + millet flours

SOS (control): composite mixture of soybeans + orange‐fleshed sweet potatoes + sorghum flour

^a^ DRI: dietary reference intake of 6‐ to 12‐month child according to World Food Program (2018).

The significant difference in calcium levels between the formulated complementary porridges and the control samples might be as a result of the incorporation of calcium‐rich ingredients including soybean, amaranth grains, and pumpkin seeds (Table 4). Moreover, the formulated complementary porridges had a significantly lower calcium content of less than 21% when compared to the DRI (260–400 mg/100 g). Findings from a Ghanaian study found that the calcium content was highest in a commercial complementary food (86.87 mg/100 g), followed by sweet potato‐based complementary food (23.91 mg/100 g) and a control (Weanimix: 1.75 mg/100 g). In that particular study, it was noted that fortification was the most probable reason for the observed high calcium content in the commercial complementary (Laryea et al., 2018). It therefore implies that there is a need to substitute the calcium deficiency in this study with other calcium‐rich foodstuffs, probably of animal origin. However, as Alam et al. (2016) put it, the only challenge would be affordability due to the low‐income status of most rural and peri‐urban households (Alam et al., 2016). Agbemafle et al. (2020) describe entomophagy (the utilization of insects as food) as one of the promising solutions for low‐income households. Their incorporation into complementary foods might offer a cheap but unreliable option due to the seasonal availability of the insects in diverse global geographical regions. Also, green leafy vegetables might be a viable option as they have been reported to contain indispensable mineral elements not only calcium but also iron and zinc, which are considered limiting minerals in complementary foods (Chacha & Laswai, 2020; Sam et al., 2018; Tumuhimbise et al., 2019).

The formulated complementary porridges had significantly higher (*p* < .05) phosphorus content when compared to the control samples. Among the formulated diets, only SAPO1, SAPO3, and SAPO5 exceeded the acceptable limits of phosphorus intake while the other diets fell below the recommended dietary reference intake (DRI) of 275 mg/100 g (at least 57% near the standard level). A previous study on complementary food formulation obtained a lower value in the range of 78.23 to 139.17 mg/100 g (Ndife et al., 2020). The main contributor of high phosphorus content in this study may be attributed to the use of phosphorus‐rich ingredients (pumpkin seeds, soybean, and amaranth) (Table 4).

With regard to zinc content, results show that all complementary porridge fell within the DRI (3–8.40 mg/100 g) according to World Food Program (2018), but in the formulated complementary porridge, it is significantly higher (*p* < .05) when compared to the control samples. Invariably, the complementary porridges can be said to be a good source of zinc since the formulated complementary porridges can provide at least 50% of zinc DRI, which is considered sufficient (Adisetu et al., 2017; Codex, 2010). Therefore, the porridges are considered suitable for use by the targeted group, which includes children and women of reproductive age. Similar to iron, zinc deficiency is also associated with stunting, anemia, and higher disease susceptibility (Agbemafle et al., 2020; Bhutta et al., 2013). It can result to permanent defects in immune function, motor, and cognitive development, as well as academic and behavioral performance (Adisetu et al., 2017; Brown, 2009).

The magnesium levels of the formulated complementary porridges were similarly significantly higher (*p* < .05) when compared to both the control samples and the DRI (54–75 mg/100 g). The higher magnesium content of formulated diets is evidently due to the raw materials utilized in complementary food formulation (Table 4). Kolawole et al. (2020) emphasized that 10% soybean, in addition to a complementary formulated food containing 10% OFSP, increased the magnesium content, indicating that soybean is a good source of the mineral. Magnesium is crucial for good infant health as it keeps the heart rhythm steady, strengthens the bones, supports a healthy immune system, and maintains normal muscle and nerve function (Ndife et al., 2020).

### 3.3 Vitamin content of the formulated porridges

The vitamins A and C content of complementary porridge ranged from 180.50 to 232.20 µg RE/100g and 1.60 to 7.80 mg/100 g, respectively (Table 6).

**TABLE 6:** Vitamin C and β‐carotene contents of the formulated complementary porridge

Sample	Vitamin C (mg/100 g)	Vitamin A (µg RE/100 g)
SOS	1.60 ± 0.01^d^	215.50 ± 0.39^c^
SMGM	2.20 ± 0.01^c^	148.50 ± 0.06**^g^**
SAPO1	1.60 ± 0.01^d^	180.50 ± 0.56**^f^**
SAPO2	2.10 ± 0.01^c^	186.40 ± 0.85**^e^**
SAPO3	4.40 ± 0.01^b^	197.10 ± 0.16**^d^**
SAPO4	7.80 ± 0.01^a^	232.20 ± 0.00^a^
SAPO5	7.50 ± 0.01^a^	224.50 ± 0.66^b^
HM^a^	16	200
HM^a^+CF	17.60–23.80	380.50–432.20
DRI^b^ (6–12 months)	30–50	400–500

Note

Values are means ± standard deviation of duplicate determinations. Means with common superscripts in the same column do not differ significantly (*p* > .05).

**Keys:** SAPO: composite mixture of soybeans + amaranth grains + pumpkin seeds + orange‐fleshed sweet potato

SMGM (control): composite mixture of soybeans + maize + groundnuts + millet flours

SOS (control): composite mixture of soybeans + orange‐fleshed sweet potatoes + sorghum flours.

Abbreviations: CF, complementary food; HM, human milk.

^a^ Published references of Campos et al. (2010).

^b^ DRI: dietary reference intake of 6‐ to 12‐month child according to World Food Program (2018).

The porridges of formulated complementary flours (SAPO1–SAPO5) had the vitamin C content ranging from 1.60 to 7.80 mg/100 g while the reference (control) samples (SOS and SMGM) recorded the vitamin C contents of 1.60 mg/100 g and 2.20 mg/100 g, respectively. However, the vitamin A content of the complementary porridge (SAPO1–SAPO5) ranged from 180.50 to 232.20 µg RE/100 g while the reference samples (SOS and SMGM) had a vitamin A content of 215.50 µg RE/100 g and 148.50 µg RE/100 g, respectively. The presence and adequacy of vitamins A and C in complementary foods cannot be overemphasized. Generally, vitamins are important nutrients that are essential for optimal health across the life cycle (Hill, 2020).

Results show that SAPO4 had a higher vitamin C content among the formulated complementary porridges though not significantly different (*p* > .05) from SAPO5, while SAPO1 recorded the least vitamin C content. The higher vitamin C in SAPO4 and SAPO5 could be due to the increased amount of orange‐fleshed sweet potato flour in its formulation. Besides dietary fiber and essential minerals, orange‐fleshed sweet potato is a rich source of vitamin C in the form of ascorbic acid (Korese et al., 2021; Van, 2000). Specifically, vitamin C is a water‐soluble micronutrient that plays a vital role in an infant's physiological functions such as facilitating collagen production, maintaining a healthy immune system, and enhancing iron absorption (Hill, 2020; Ofoedu, Iwouno, et al., 2021). Unlike many micronutrients, vitamin C is unique because of its antioxidant capacity, which helps protect cells from free radical damage (Ofoedu, You, et al., 2021). In this study, though porridges from complementary flours may be considered as a poor source of vitamin C when compared to the recommended dietary reference intake (DRI) of 30–50 mg/100 g according to WFP (2018), it is higher than the values of 0.64–0.94 mg/100 g reported by Oyegoke et al. (2018) for complementary flour formulated from yellow maize, soybean, millet, and carrot composite flours. The variations may be attributed to the differences in the type of raw materials, processing treatments and methods, and state of the substrate (flour or porridge). However, the lower vitamin C content of the formulated complementary flour in this study when compared to the DRI could be as a result of the influence of processing treatment on the ingredients. Extrusion treatment is a processing technology that combines different unit operations such as cooking, mixing, shearing, kneading, shaping, and forming during food processing (Verma et al., 2018). As vitamin C is susceptible to heat destruction, the reduced vitamin C content in the studied complementary porridges could be a result of heat treatment from the extrusion cooking. Specifically, though the complementary porridges from SAPO4 and SAPO5 cannot meet the DRI when considered independently, at least about 78%–80% of the DRI can be met when the complementary porridges are combined with human milk as part of an infant's daily meal.

On the contrary, SAPO4 had the higher vitamin A content followed by SAPO5 while SAPO1 recorded the least vitamin A content of 180.50 µg RE/100 g. The variations could be as a result of the proportions of soybean and pumpkin seed in the formulation. Soybean and pumpkin seeds are well known as oil seeds with fat contents of 22.30% and 43.46%, respectively (Table 2), thereby suggesting adequate dissolution of lipophilic vitamins such as vitamin A (Ofoedu, Iwouno, et al., 2021). Moreover, vitamin A is a fat‐soluble micronutrient that is crucial for rapid growth and fighting infections (UNICEF, 2019), as its insufficiency has been implicated to be the leading cause of visual impairment (night blindness) and high risk of deaths from common illness in children such as diarrhea (Ofoedu, Iwouno, et al., 2021). In this study, complementary porridges with the higher vitamin A content (SAPO4 and SAPO5) had the least amount of soybean and pumpkin seeds while formulations with higher amounts of soybean and pumpkin seeds had the least vitamin A content in their porridges. This could be a result of thermal degradation of the vitamin A components in porridges of SAPO1–SAPO3 by heat from extrusion cooking and porridge preparation while a greater percentage of the vitamin A components engulfed in the lipid content of SAPO4 and SAPO5 may be encapsulated by starch molecules and then protected/shielded from heat as a result of increased amount of amaranth grains and orange‐fleshed sweet potato flour (Kapusniak & Tomasik, 2006). Additionally, besides the proportions of soybean and pumpkin seeds used in the formulation, the increased amount of orange‐fleshed sweet potato could also influence the higher vitamin A content in SAPO4 and SAPO5. Previous studies have shown that orange‐fleshed sweet potato (OFSP) is a rich source of vitamin A in the form of β‐carotene (Burri, 2011; Korese et al., 2021; Van, 2000). This corroborates the findings of Waized et al. (2015), which reported that in most OFSP species, a small amount of only 125 g could provide an adequate amount of vitamin A commended on a day‐to‐day basis for both children and lactating mothers. Just like in vitamin C, the vitamin A content of the porridges was lower than the DRI of vitamin A (400–500 µg RE/100 g). Importantly, relative to previous studies on indigenously made complementary foods, results show that the vitamin A content of complementary porridge in this study is generally higher than that in the control samples and the value of 98.21 µg RE/100 g reported by Jemberu et al. (2016) in a complementary porridge formulated using maize with orange‐fleshed sweet potato and bean flour. The variations could be as a result of the difference in ingredients used for the formulation. Similarly, the vitamin A content in the complementary porridge cannot meet the DRI when considered singly, but an appreciable number of the complementary porridges (especially SAPO4 and SAPO5) can meet the DRI when the porridge is combined with human milk as part of an infant's daily meal (Table 6).

### 3.4 Energy and nutrient density of the formulated porridges

The energy and nutrient density of complementary porridge is presented in Table 7.

**TABLE 7:** Energy and nutrient density of the formulated complementary porridge

Sample	Energy density (Kcal/g)	Protein density (g/100 Kcal)	Vitamin A density (µg RE/100 Kcal)	Iron density (mg/100 Kcal)	Zinc density (mg/100 Kcal)
SOS	1.95^e^	0.34^e^	56.56^a^	4.83^b^	2.49^b^
SMGM	1.90^f^	0.38^e^	40.00^e^	6.52^a^	3.04^a^
SAPO1	2.58^a^	1.05 ^a^	35.81^g^	3.65^c^	2.40^c^
SAPO2	1.55^g^	0.99^b^	37.51^f^	3.06^d^	1.47^e^
SAPO3	2.41^b^	0.79^c^	42.03^d^	2.86^e^	1.51^d^
SAPO4	2.39^c^	0.64^d^	49.84^c^	1.40^g^	0.88^g^
SAPO5	2.18^d^	0.75^c^	52.75^b^	2.56^f^	1.25^f^
Recommended standard^a^	≥0.8	0.7–1.0	5–31	2.4–4.5	0.5–1.6

**Keys:** SAPO: composite mixture of soybeans + amaranth grains + pumpkin seeds + orange‐fleshed sweet potato.

SMGM (control): composite mixture of soybeans + maize + groundnuts + millet flours

SOS (control): composite mixture of soybeans + orange‐fleshed sweet potatoes + sorghum flours

^a^ Published references of WHO/UNICEF (1998) and WHO (2002).

As shown in Table 7, the energy density of complementary porridges was in the range of 1.55–2.58 Kcal/g, having a significant difference (*p* < .05) among the values. However, porridges from the formulated complementary food were significantly higher (*p* < .05) than the control (SOS and SMGM) samples. Results show that the energy density of the complementary porridge met the minimum recommended daily energy requirement of ≥0.8 Kcal/g reported by WHO/UNICEF (1998) and WHO (2002) for the targeted infant group. Multiple studies have reported different energy densities of complementary foods such as 0.30 Kcal/g for a fermented millet complementary porridge, 0.35 Kcal/g for sorghum porridge, 0.39 Kcal/g for maize porridge, 0.50 Kcal/g for maize and crayfish porridge, and 0.60 Kcal/g for maize and soybean porridge (Mouquet‐Rivier et al., 2008; Ogbonnaya et al., 2012; Oladiran & Emmambux, 2020). The variations in energy densities of previously mentioned studies, when compared to the current study, might be as a result of the difference in raw materials (ingredients) and the processing methods. In this study, soybean and pumpkin seeds with high‐fat content of 22.30% and 43.46%, respectively (Table 2) were utilized as ingredients during the development of the complementary foodstuffs. According to a report by World Health Organization (WHO, 2009), fat is an integral component of complementary food formulation because they increase the energy density of foods, enhance absorption of vitamin A and other fat‐soluble vitamins, and make the food taste better. On the contrary, the lower energy densities recorded in a previous work could be due to the amount of water added to the complementary porridge to make it less viscous, thereby diluting the porridge and causing energy thinning of the complementary porridge (Amagloh et al., 2013). The energy density of complementary food is well known as a determinant of the quantity of food needed to meet the energy needs of infants (Oladiran & Emmambux, 2020). In other words, while a large amount of a low energy‐dense foodstuff is required for complementary feeding, a smaller amount of an energy‐dense foodstuff would be necessary to be fed to an infant.

The formulated complementary porridge (SAPO1–SAPO5) had a protein density ranging from 0.64 to 1.05 g/100 Kcal while the reference (control) samples (SOS and SMGM) recorded a protein density of 0.34 g/100 Kcal and 0.38 g/100 Kcal, respectively (Table 7). The protein density of the formulated complementary porridge is significantly higher (*p* < .05) than in the reference samples. Importantly, the overall effect on protein quality is dependent on ingredient composition and the processing treatments used, as germination and extrusion conditions may have influenced the properties of the complementary porridge. Germination has been reported to increase the protein quality of food (Ofoedu, Akosim, et al., 2021; Ofoedu et al., 2019, 2020; Okafor et al., 2018; Osuji et al., 2019, 2020). Similarly, extrusion cooking has also been demonstrated to increase lysine availability and protein digestibility (via reduction of antinutritional factors) due to the unfolding of protein molecules (Aryee et al., 2018). Low protein density in complementary food suggests that such food may not be able to supply all the required essential amino acids in the right proportion, thus resulting in food with low protein quality.

The micronutrient (vitamin A, iron, and zinc) density of complementary porridge ranged from 35.81 to 56.56 µg RE/100 Kcal for the vitamin A density, 1.40 to 3.65 mg/100 Kcal for iron density, and 0.88 to 2.40 mg/100 Kcal for zinc density (Table 7). The micronutrient densities of the reference samples were significantly higher (*p* < .05) than those of the formulated complementary porridges except for the vitamin A density of SMGM. Furthermore, results show that all the complementary porridge met the minimum recommended requirements for vitamin A, iron, and zinc densities according to WHO/UNICEF (1998) and WHO (2002). In this study, the vitamin A density of complementary porridge is slightly higher than the values of 24.55–42.81 µg RE/100 Kcal reported by Tenagashaw et al. (2017). The variations might be attributed to differences in processing raw materials. Results show that SAPO4 and SAPO5 have a higher vitamin A density than the other formulated diets. Specifically, orange‐fleshed sweet potato, which is known to be a rich source of β‐carotene, is in higher proportion in the formulation recipe of SAPO4 and SAPO5. In addition, high starch‐containing amaranth grain and sweet potato flours used in this formulation may have engulfed the lipid content of SAPO4 and SAPO5, thereby shielding it from thermal degradation resulting from extrusion cooking (Kapusniak & Tomasik, 2006). Lipids are known to enhance the absorption of vitamin A and other fat‐soluble vitamins (WHO, 2009) and also enhance the encapsulation of other solid components in food (Ozkan et al., 2020).

The complementary porridge densities of iron and zinc, which are considered “problematic” nutrients in children and infants’ food in developing nations, were met, except for SAPO4 (Table 7). Thus, this study proposes that the complementary porridges are good sources of iron and zinc. The considerably high iron density in the formulated complementary porridge could be due to the significant reduction in antinutrients that are potential iron inhibitors such as phytates, by extrusion cooking. Additionally, the concentration of vitamin C may be such that enhanced iron absorption, in which case the complementary porridges are considered to have iron bioavailability (Ruel et al., 2004). Zinc is particularly an important nutrient for the growth and immune function of infants and young children. Just like iron, the amount of zinc in breastmilk is low after the infant is about 6 months of age. Unlike many indigenous complementary porridges, the formulated diet in this study is a typical candidate for zinc, as the porridges were able to meet the minimum requirement of zinc density according to WHO/UNICEF (1998) and WHO (2002). However, there seems to be a positive correlation between iron density and zinc density. According to Brown and Lutter (2000), diets that are high in bioavailable iron are also likely to be high in bioavailable zinc, as both micronutrients are contained in similar foods.

Furthermore, Table 8 briefly shows the compliance or noncompliance of the formulated complementary porridges to recommended standard levels.

**TABLE 8:** Distribution summary of formulated complementary porridges' compliance to recommended standards

Sample	Energy and nutrient densities					Vitamins		Minerals						Percentage compliance (%)
	Energy	Protein	Vit. A	Fe	Zn	Vit C	Vit A	Na	Fe	Ca	P	Zn	Mg	
SAPO1	+	+	+	+	+	−	−	−	+	−	+	+	+	69.23
SAPO2	+	+	+	+	+	−	−	−	+	−	−	+	+	61.54
SAPO3	+	+	+	+	+	−	−	−	+	−	+	+	+	69.23
SAPO4	+	+	+	−	+	−	+	−	+	−	−	+	+	61.54
SAPO5	+	+	+	+	+	−	+	−	+	−	+	+	+	76.92

**Keys:** +: met the recommended standard level or fell within the acceptable limit.

–: Failed to meet the recommended standard level or fell outside the acceptable limit.

Overall, all the formulated complementary porridges were able to meet the stipulated standards of energy and nutrient (protein, vitamin A, zinc, and iron) densities except for SAPO4 that could not meet the recommended standard of iron density. Although SAPO4 met the recommended vitamin A acceptable limit, it could not meet that of phosphorus. While none of the formulated diets met calcium and the vitamin C content, all of them exceeded the acceptable limit of sodium. From the results, SAPO5 has the highest percentage compliance (76.92%), which is indicative of its high promise in offering the limiting nutrients required in complementary diets. Therefore, SAPO5 porridge can serve as a key diet for adequate complementation of breastfeeding among other formulated complementary porridges.

## 4 CONCLUSION

In this study, the nutritional composition of the formulated complementary porridges evaluated showed that a good and acceptable complementary diet can be produced from blends of soybean, orange‐fleshed sweet potato, amaranth grains, and pumpkin seed flours. The formulated complementary porridges exhibited a considerable level of compliance to recommended standards based on the targeted nutrients of interest. Results showed that the germination process and extrusion cooking influenced the nutritional composition of complementary porridges to a great extent by increasing the protein density, iron availability, and zinc availability, while the vitamin A density, the vitamin A content, and the vitamin C content were decreased. Furthermore, changes in the proportion of ingredient‐mix (blend ratio) significantly influenced the energy and nutrient densities of the formulated complementary porridges. Specifically, SAPO5 porridge with about 76.92% compliance level holds a high promise in providing the needed energy and protein densities, as well as the required micronutrient (vitamin A, zinc, and iron) densities that are limited in most locally made complementary porridges in sub‐Saharan Africa. Since the results of this study have revealed the significant contribution of the developed product to the nutritional requirements of children aged 6–12 months, it is therefore recommended that utilization of soybeans, amaranth grains, pumpkin seeds, and orange‐fleshed sweet potato in complementary feeding should be promoted to our communities and other developing nations at large. Also, as one of the strategies of improving the energy, protein, vitamin A, iron, and zinc in complementary foods used in Tanzania, education on the nutrition benefits of these foods in complementary feeding should be provided to all caregivers and the community as a whole.

## CONFLICT OF INTEREST

The authors declare no conflict of interest.

## AUTHOR CONTRIBUTION

**Mary Raphael Marcel:** Conceptualization (equal); Data curation (equal); Funding acquisition (lead); Investigation (equal); Methodology (equal); Resources (equal); Software (equal); Validation (equal); Writing – original draft (equal). **James Simon Chacha:** Project administration (equal); Supervision (equal); Visualization (equal); Writing – original draft (equal); Writing – review & editing (equal). **Chigozie Emmanuel Ofoedu:** Project administration (equal); Supervision (equal); Validation; Visualization (equal); Writing – original draft (equal); Writing – review & editing (equal).

## ETHICS APPROVAL

The study does not involve any human or animal testing.

## Data Availability

The data that support the findings of this study are available from the corresponding author upon request.
