# Erratum to Nutritional evaluation of complementary porridge formulated from orange‐fleshed sweet potato, amaranth grain, pumpkin seed, and soybean flours

**DOI:** 10.1002/fsn3.2801

**Published:** 2022-03-04

Tables 3, 5, 6, and 7 were originally published showing asterisks to represent the table footnotes instead of superscript letters. This has now been corrected.

The publisher apologizes for this error.
